# Imputed whole genome sequence based association pinpoints genetic architecture of labour dystocia of pigs using multi-breed populations

**DOI:** 10.1186/s12711-026-01076-3

**Published:** 2026-08-09

**Authors:** Shenping Zhou, Jinyi Han, Jingli Yuan, Chuanmin Qiao, Zhe Chao, Dongdong Duan, Mengyu Li, Zhenyu Wang, Shuyi Tan, Xinjian Li, Wenshui Xin

**Affiliations:** 1 Sanya Institute, Hainan Academy of Agricultural Sciences, Sanya, 572025 Hainan People’s Republic Of China; 2 Institute of Animal Science and Veterinary Medicine, Hainan Academy of Agricultural Sciences, Haikou, 571100 People’s Republic Of China; 3 National Key Laboratory for Swine Genetic Improvement and Germplasm Innovation, Jiangxi Agricultural University, Nanchang, 330045 People’s Republic Of China

## Abstract

**Background:**

Labour dystocia (LAD) in sows is a common reproductive disorder that reduces piglet survival rates and increases the number of stillbirths, causing substantial economic losses to pig farms. However, the genetic basis of LAD remains elusive. Herein, we performed a single-breed genome-wide association study (GWAS) for LAD using imputed whole-genome sequence data from 3263 sows (487 Landrace and 2776 Yorkshire).

**Results:**

In this study, analysis of the reproductive traits showed that the total number of piglets born and the number of piglets born alive were 1.43–2.85 and 1.60–2.94 higher, respectively, in sows with natural labour than in those with LAD. We identified 250 and 12 SNPs associated with LAD in primiparous and multiparous sows, respectively. Furthermore, in primiparous Yorkshire sows, two major QTL were fine-mapped to a 498.44 kb interval (23.18–23.68 Mb) on SSC2 and a 779.85 kb interval (43.34–44.12 Mb) on SSC9. Further analysis revealed that the two significant SNPs (2_23340612 and 9_44119733) within these QTL regions were located within regulatory regions across multiple pig breeds and tissues. The potentially regulatory SNP (2_23340612) is located within the binding sites of 27 transcription factors, that are primarily involved in oxytocin signalling, muscle contraction, and the development of female genitalia and embryo. The other potentially regulatory SNP (9_44119733) is located within the binding sites of eight transcription factors, among which three transcription factors (ZNF274, HMGA1, and CTCF) are predicted to interact with six candidate genes (*APOA1*, *APOC3*, *APOA4*, *APOA5*, *BUD13*, and *ZPR1*). Finally, eight promising candidate genes (*APOA1*, *APOC3*, *APOA4*, *APOA5*, *BUD13*, *ZPR1*, *ACAN*, and *HAPLN3*) were identified to be associated with LAD. Functional enrichment analysis indicated that the candidate genes were enriched in pathways related to lipid metabolism, extracellular matrix organization, cell adhesion and response to estrogen.

**Conclusions:**

The LAD is a prominent reproductive disorder trait that negatively impacts the reproductive efficiency of sows. This study identified potentially regulatory SNPs (2_23340612 and 9_44119733) and eight promising genes (*APOA1*, *APOC3*, *APOA4*, *APOA5*, *BUD13*, *ZPR1*, *ACAN*, *HAPLN3*) associated with LAD. To our knowledge, this is the first whole-genome-sequence-based GWAS with large-scale reproductive data to identify genetic markers and candidate genes of LAD of sows.

**Supplementary Information:**

The online version contains supplementary material available at 10.1186/s12711-026-01076-3.

## Background

The number of piglets born alive (NBA) is an important indicator of the reproductive efficiency and is influenced by many factors, including labour dystocia (LAD). The LAD in sow refers to piglet birth intervals more than 45 min, often requiring obstetric assistance [1, 2], while normal delivery is when a sow expels all the foetuses and placentas without obstetrical intervention [3]. The LAD reduces the NBA as a result of increasing the stillbirth rate and negatively impairs the health and reproductive efficiency of sows. After decades of intensive genetic improvement, the litter size of domestic pigs has increased remarkably, which has been accompanied by longer farrowing duration and a higher ratio of stillbirths. For example, the duration of delivery increased from approximately two hours per 12 piglets to six hours and 40 min per 19 piglets born, leading to a rise in the incidence of LAD in sows from 0.25% to 5% and an increase in the number of stillbirths from 0.7 to 1.2 [3–7]. Therefore, understanding the genetic mechanism of parturition in sows helps prevent LAD and reduce the number of stillbirths. However, there are few studies on parturition in sows, probably due to the low frequency of LAD in the past. To date, a total of 56,615 QTLs have been reported on the Pig QTLdb (https://www.animalgenome.org/cgi-bin/QTLdb/SS/index, accessed on 30 March 2025). Among them, 7338 QTLs are associated with reproductive traits; however, there are no published QTLs and genes found for LAD in pigs.

Sow parturition is a biological process affected by many factors, including uterine contractions [8], parity [9], and piglet size [10]. A genome-wide association study (GWAS) is an efficient tool for dissecting the genetic architecture of complex traits and has accelerated genetic improvement in pigs. For instance, Ren et al. [11] found that *PPARD* is the causal gene affecting pig ear size by GWAS. Ma et al. [12] performed GWAS for glycolytic potential (GP) in pigs and identified *PHKG1* as a causal gene affecting glycogen content in skeletal muscle. Moreover, Cho et al. [13] conducted GWAS for meat colour (a*) and intramuscular fat in pigs, revealing that the *MYH3* gene affects muscle fibre-type composition and intramuscular lipid content. Although GWAS has successfully identified causal genes for several economically important traits in pigs, many remain unknown, which is related to the statistical power of the studies. It is widely known that the power of GWAS for the traits with complex and low heritability depends on the marker density and sample size. In addition, the number of SNPs in the whole genome of pigs is as high as hundreds of millions, while common commercial SNP chips are relatively sparse, with only 50k to 80k SNPs [14–18], resulting in sparse marker coverage, which leads to wide QTL intervals and low fine-mapping resolution. The imputation method that imputes low-density SNP sets to the whole genome sequence using a re-sequenced reference is a cost-effective way to obtain a high-density marker panel and increase the power of GWAS, thereby identifying key genes and variants that affect complex traits [14]. As such, it offers a solution to the prohibitive cost problem of applying whole-genome resequencing to large-scale populations, standing as a cost-effective alternative for deriving high-density genetic variants [19, 20].

The LAD is a kind of reproductive disorder of sows, but its genetic basis is still poorly known. In this study, we analysed LAD and applied imputation to obtain the imputed whole-genome sequences of 3263 sows and then performed GWAS for LAD. To the best of our knowledge, this is the first GWAS of LAD in pigs. The objectives of this study are: (I) to identify key genes and variants that affect LAD, (II) to dissect the genetic architecture of LAD in pigs.

## Methods

### Animals and phenotypes collection

Between 2018 and 2023, reproductive phenotype data were collected for the top four parities from 6960 sows (1,196 Landrace and 5,764 Yorkshire). These animals were raised at Henan Xinda Animal Husbandry Co., Ltd. (Henan, China), which also provided all phenotypic data. During gestation, sows were housed in gestation units at room temperature (18–20 °C). The sows were fed a commercial gestating diet at 2.0–2.5 kg/day during early gestation (days 0–88) and at 3.0–3.5 kg/day during late gestation (days 89–110), in equal portions three times a day. Sows were transferred from the gestation units to the farrowing rooms 7.0 ± 3.0 days prior to the expected farrowing date. The temperature of the farrowing rooms was strictly controlled at 20 °C, and the breeders took turns inspecting the rooms for 24 h per day. Obstetric intervention was initiated if a sow that had delivered one or more piglets ceased farrowing for over 45 min, or if a sow showed signs of labour onset but failed to deliver any piglets within 45 min. In addition, the reproductive performance of the sow was recorded at delivery, including labour natural (LAN) and LAD, total number born (TNB), NBA, etc. LAN represents a sow of normal delivery, and LAD represents a sow requiring obstetric intervention.

To assess the LAD recurrence rate, sows with a history of LAD and delivery records from the previous four parities were selected. Subsequently, the proportion of sows with two or more abnormal deliveries in the LAD sows was statistically analyzed. To investigate the effect of delivery status on the reproductive performance in sows, a one-way ANOVA was performed using R software to compare 6960 sows with LAN and LAD. Furthermore, ear samples of 3263 randomly selected sows (487 Landrace and 2776 Yorkshire sows) from the aforementioned 6960 sows were collected for follow-up genotyping.

## Genotype and imputation

The genomic DNA of 3263 sows was extracted by standard Trizol DNA extraction methods. DNA integrity was assessed by electrophoresis and ultraviolet spectrophotometry. Concentrations of all DNA samples were diluted to 50 ng/µL. The diluted DNA samples were sent to COMPASS BIOTECHNOLOGY (Beijing, China) for genotyping of the Porcine 50 K SNP chip. Raw genotype data of the 3263 individuals were quality controlled (QC) using PLINK v1.9 [21]. The following criteria were used to filter SNPs: animal data with call rates < 95%, SNPs with call rates of < 90%, with minor allele frequency < 1%, and *P*-value < 1.00E-06 for the Hardy-Weinberg equilibrium test, were excluded. In total, 41,756 SNPs and 3263 individuals were retained for imputation analysis.

Beagle version 5.2 software was used to perform genome-wide phasing and imputation with the default parameters partially [22], except that the parameter Ne (effective population size) was set to 210 (according to our unpublished data). The parameter window was set to 80 cM, and overlap was set to 70 cM. The imputation reference panel was constructed by combining publicly available whole-genome sequence data from 1,874 pigs across 43 breeds (average sequencing depth: 17.1×) [23] with whole-genome resequencing data from 819 Yorkshire and 144 Landrace pigs (average sequencing depth: 10.0×) generated in this study. After imputation, quality control was performed using the same criteria as for the chip-derived genotypes, and only SNPs passing QC were retained for subsequent analyses. Imputation accuracy was then assessed using the squared DR value; across all autosomes, the per-chromosome accuracy ranged from 0.889 to 0.941 [Additional file 1, Fig. S1]. The numbers of cases and controls for primiparous and multiparous sows in each breed, as well as the final number of effective SNPs after quality control [Additional file 2, Table S1]. Notably, the primiparous and multiparous records were obtained from the same cohort of sows.

## Population structure analysis

Population stratification can cause false-positive associations and thus potentially affect GWAS reliability. Therefore, software R [24] and GCTA [25] were used to evaluate the population structure of the two sow populations (Landrace and Yorkshire). Accordingly, principal component analysis (PCA) was used to evaluate the similarity of the genetic background in two sow populations by GCTA v1.92.4beta. Quantile-quantile (Q-Q) plots were used to assess the reliability of GWAS results using the R v4.3.2 software.

## Genome-wide association studies

**Fig. 1:**
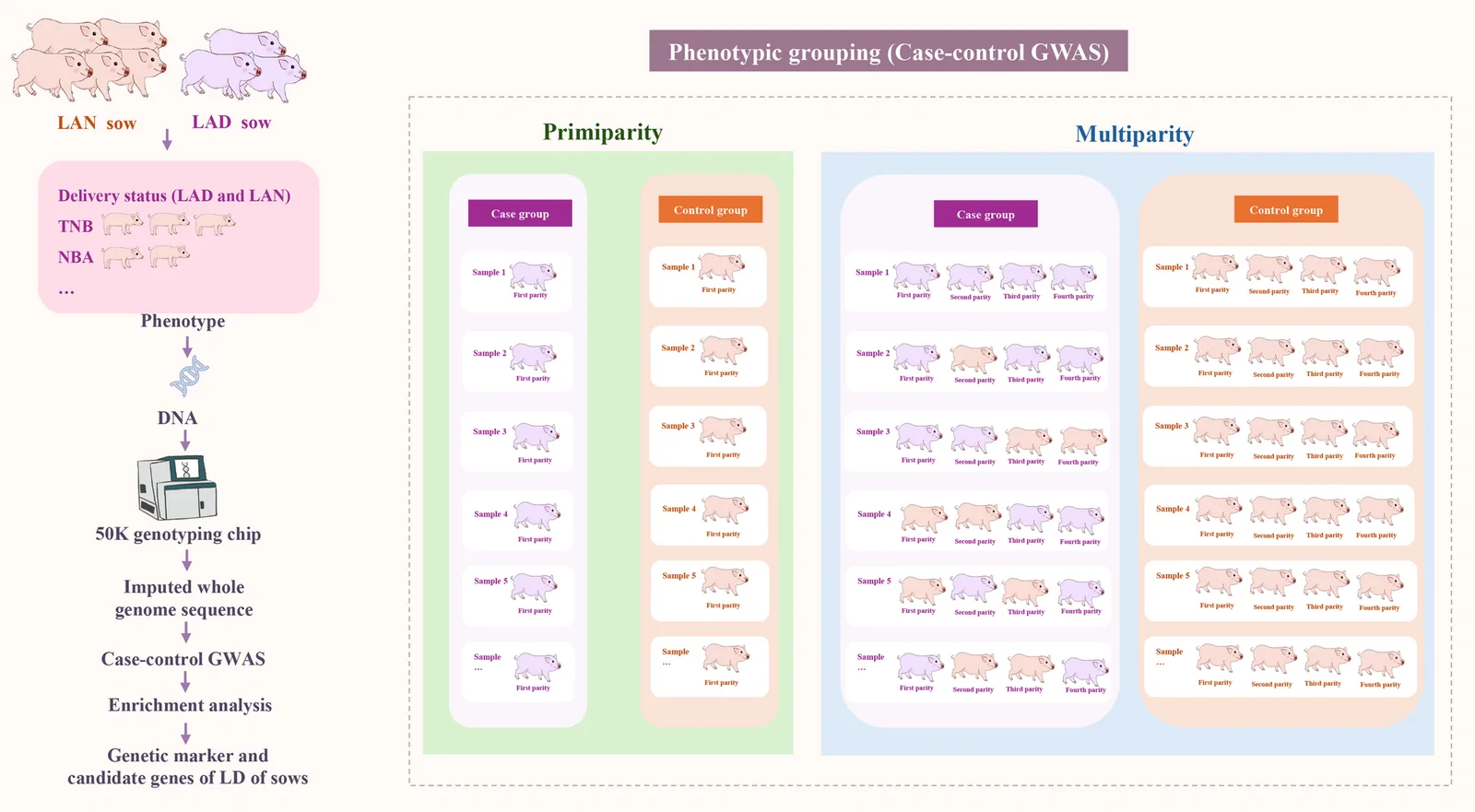
Technical route diagram. Labour dystocia (LAD); Labour natural (LAN); Total number born (TNB); Number born alive (NBA)

The case-control GWAS was performed using a univariate linear mixed model in the GEMMA v0.98.1 software, and the same univariate linear mixed model was used for analysing the single-breed and multi-breed [26–29]. As shown in Fig. 1, we performed GWAS for LAD separately in primiparous sows and multiparous sows. For primiparous sows, those sows with LAD were defined as cases, while those with LAN served as controls. For multiparous sows, those with two or more LAD in their previous four parities were classified as cases, whereas those with LAN in all four parities served as controls. The specific grouping information of different parities was shown in [Additional file 2, Table S1]. The following univariate mixed linear model was used to test the association between variants and LAD:

$$\:\mathbf{y}=\mathbf{X}\boldsymbol{\upalpha\:}+\mathbf{x}\boldsymbol{\upbeta\:}+\mathbf{u}+\boldsymbol{\varepsilon\:},$$

$$ {\mathbf{u}} \sim {\mathrm{MVN}}_{{\mathrm{n}}} \left( {0,\,{{\upsigma }}_{{\mathrm{u}}}^{2} {\mathbf{G}}} \right); \boldsymbol{\varepsilon} \sim{\mathrm{MVN}}_{{\mathrm{n}}} \left( {0,\upsigma}_{{\mathrm{e}}}^{2} \mathbf{I}_\mathrm{n} \right)$$

n is the number of individuals; **y** refers to the *n* × 1 vector of phenotypic values for the LAD trait; **X** is the n × c (c is the number of covariates) incidence matrix of covariates (Primiparous sows: the top three eigenvectors of PCA, year-season, batch; Multiparous sows: the top three eigenvectors of PCA); **α** is the c × 1 vector of fixed effects; **β** is the fixed effect of the SNP; **x** is an *n* × 1 vector of SNP genotypes; **u** is a *n* × 1 vector of random effects; **ε** corresponds to an *n* × 1 vector of errors; σ^2^_u_ and σ^2^_e_ represent the genetic and residual variances, respectively; **G** is a n × n genomic relatedness matrix was estimated by GEMMA; **I**_n_ is an n × n identity matrix and MVN is multivariate normal distribution.

Based on the results of GWAS chip data, most studies adopted the Bonferroni correction method to set the significance threshold. However, the Bonferroni correction is rather strict for the results of GWAS based on imputed genotype data [30, 31]. Given the assumption that an equal number of independent haplotype segments between pigs and humans is held, it is suggested that 5.00E − 08 could serve as a genome-wide significant threshold in pig GWASs [32, 33]. Thus, in the current study, thresholds for genome-wide significance and suggestive significance were set at 5.00E − 08 and 1.00E − 06, respectively.

## Estimation of genetic parameters and the explained phenotypic variance

In this study, the GREML-LDMS method in GCTA software was used to estimate the genomic heritability of LAD in sows [25, 34]. Estimation of genetic correlation between LAD of different parities sows was performed using bivariate GREML (also computed using GCTA). The BLUPF90 software [35] was used to estimate genetic correlation between LAD and reproductive traits.

### Statistical fine mapping for the QTLs on SSC2 and SSC9

Bayesian fine-mapping was performed using FINEMAP v1.4.1 with the Shotgun Stochastic Search (SSS) algorithm [36]. For the QTL on SSC2, all SNPs surpassing the suggestive significance threshold in the GWAS were included in the fine‑mapping analysis. For the QTL on SSC9, only four SNPs exceeded this threshold. Therefore, fine‑mapping was extended to all SNPs within the 2‑LOD drop‑off interval of the top SNP. GWAS summary statistics (effect sizes and standard errors) and the within‑sample LD correlation matrix (r^2^) were used as input. The maximum number of causal SNPs was set to 5 (--n-causal-snps 5). All analyses were based on GWAS results from the Yorkshire primiparous sows. Posterior inclusion probabilities (PIP) and 95% credible sets were reported.

## Motifs and transcriptional factor determination

To investigate whether the potentially regulatory SNP is located within transcription factor binding sites (TFBS), we extracted DNA sequences spanning ± 100 bp (a total of 201 bp) around the mutation site. These flanking sequences were used as input to the MEME Suite v5.5.7 online tool (https://meme-suite.org/meme/tools/meme) to predict potential motifs [37]. The MEME Suite is a computational tool designed to identify one or more motifs in DNA or protein sequences, which may represent potential TFBS. Subsequently, the predicted motifs were submitted to the TOMTOM online tool (part of the MEME Suite) to identify candidate transcription factors through motif comparison against the JASPAR CORE (2026) vertebrates database. Among the predicted transcription factors, priority was given to those whose binding motifs directly encompass the potentially regulatory SNP site, as a variant located within the core binding motif is more likely to affect the physical interaction between the transcription factor and the DNA.

## Significant variant annotation and enrichment analysis

In this study, all significant variants were annotated using the Ensembl Variant Effect Predictor (VEP) (Variant Effect Predictor - *Sus scrofa* 11.1 assembly - Ensembl genome browser 112) [38]. To determine whether these variants reside within functional regulatory regions, ChIP‑seq (H3K27ac, H3K4me3) and ATAC‑seq peak data from various pig breeds and tissues were obtained from the publicly available GEO database. Overlap between the significant SNPs and the downloaded peaks was then assessed using BEDTools [39]. Enrichment analysis of the candidate genes and predicted transcription factors was performed using the KOBAS 3.0 online tool (http://kobas.cbi.pku.edu.cn/) [40]. The statistical significance of enriched terms was calculated by Fisher’s exact test with FDR correction (*P* < 0.05) [41]. The interaction network analysis of candidate genes and transcription factors was conducted by STRING software v12.0 (http://string-db.org).

## Results

### Phenotype statistics

**Fig. 2:**
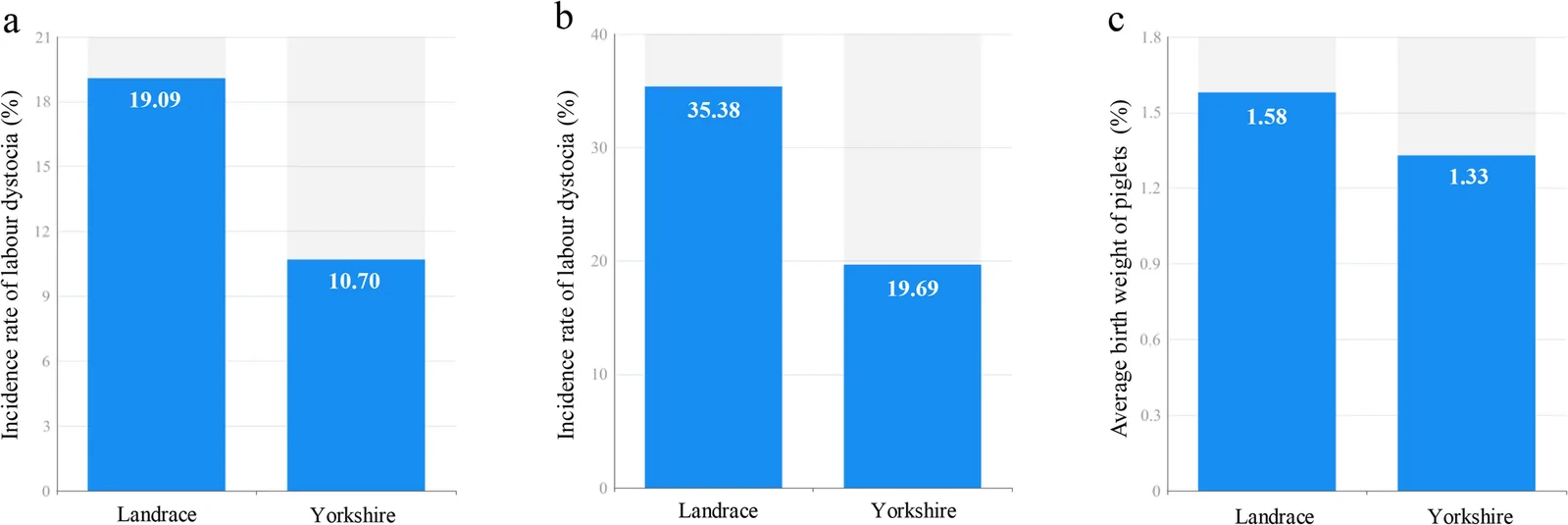
Statistics of phenotypic data. **a** Incidence rate of LAD in different populations; **b** the proportion of sows with two or more abnormal deliveries in the LAD sows; **c** average birth weight of piglets in different populations

This study analysed the incidence of LAD in the previous four parities of Landrace and Yorkshire sows. As presented in Table 1, the incidence of LAD in the first to fourth parities ranged from 8.97% to 23.41% and from 8.97% to 12.03% in Landrace and Yorkshire sows, respectively. In addition, the incidence of LAD in first-parity sows was higher (Landrace: 23.41%; Yorkshire: 12.03%) than in subsequent parities (Landrace: 8.97–20.47%; Yorkshire: 8.97–10.91%) (Table 1). In the previous four parities, the incidence of LAD was 19.09% in Landrace and 10.70% in Yorkshire (Fig. 2a). Interestingly, after a first LAD case, the probability of LAD reoccurrence in sows was 35.38% (Landrace), and 19.69% (Yorkshire) (Fig. 2b). Furthermore, the average birth weights (ABW) of piglets born from Landrace and Yorkshire sows was 1.58 kg and 1.33 kg, respectively (Fig. 2c). These results showed that parity, breed, and piglet size may affect sow delivery.

**Table 1:** Phenotypic statistics on the incidence of LAD in sows

Population	Parity	Number	Number of LAD	Incidence rate of LAD (%)
Landrace	1	487	114	23.41
	2	342	70	20.47
	3	246	35	14.23
	4	145	13	8.97
Yorkshire	1	2776	334	12.03
	2	2173	195	8.97
	3	1659	181	10.91
	4	1108	116	10.47

**Fig. 3:**
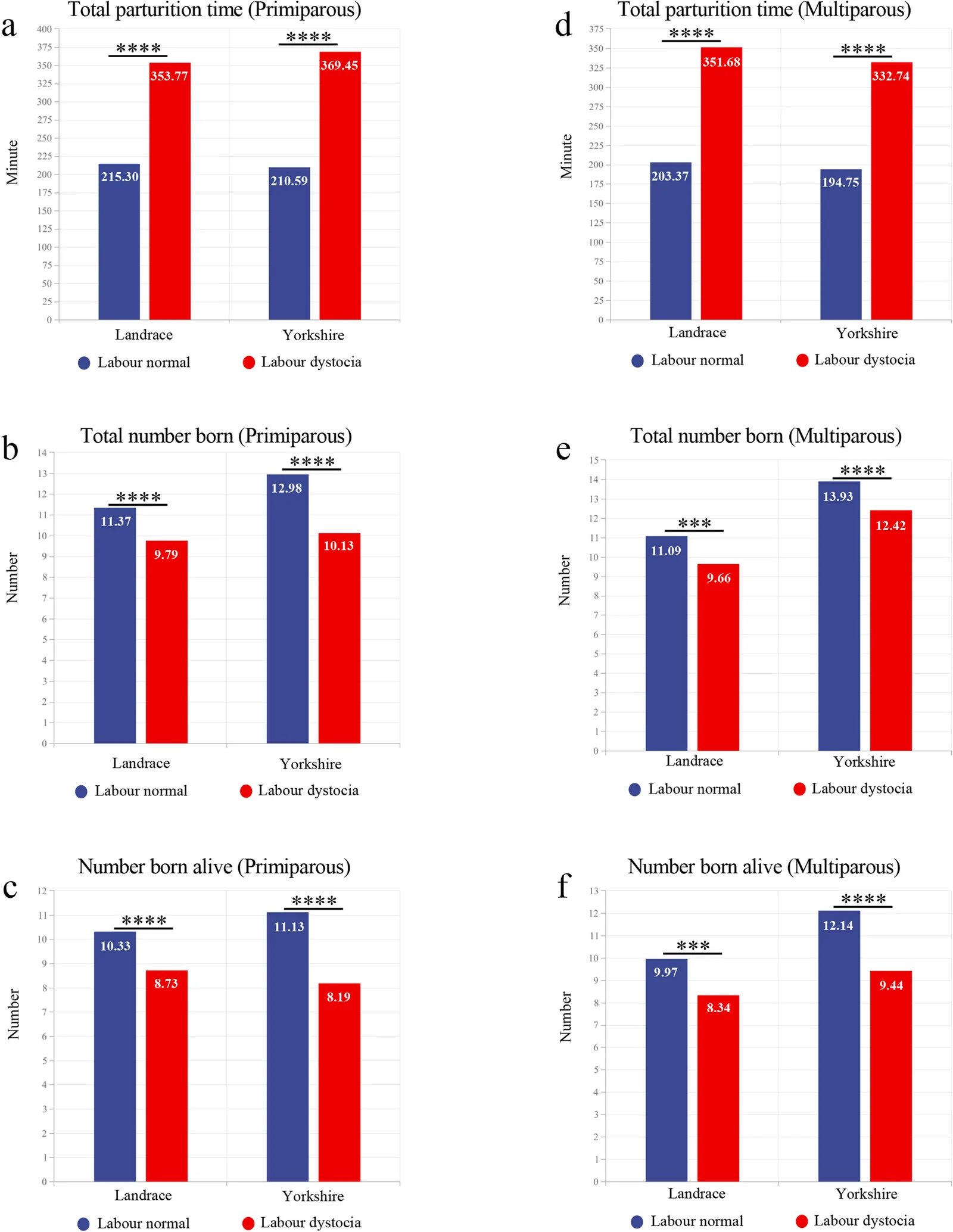
One-way ANOVA for reproductive performance in sows. Total number born (TNB); Number born alive (NBA); Total parturition time (TPT). *P* < 0.001 (***); *P* < 0.0001 (****)

In Yorkshire sows, the genetic correlation coefficient of LAD between primiparous and multiparous sows was 0.88 (Table 2). In addition, the genomic heritability of the LAD trait was low to moderate (from 0.11 to 0.19) in the Landrace and Yorkshire sows (Table 2). To further analyze the effects of LAD on the reproductive traits of sows, this study assessed the genetic correlation between LAD and the reproductive traits of sows. As presented in [Additional file 3, Table S2], the genetic correlation coefficients between LAD and TNB, as well as between LAD and NBA, were − 0.77 and − 0.89, respectively. The results showed that there was a strong negative genetic correlation between LAD and reproductive traits in sows. In addition, this study also used one-way ANOVA to compare the reproductive performance of the 3263 sows with LAN and LAD, respectively. The results were shown in Fig. 3, in the primiparous and multiparous sows of the two breeds, the TNB and NBA of the LAN sows were significantly higher than those in the LAD sows (*P* < 0.001). The TPT of the LAN sows was significantly lower than those in the LAD sows (*P* < 0.001). In the primiparous sows of the two breeds, the number of TNB and NBA of sows with LAN were 1.58–2.85 and 1.60–2.94 more than those with LAD, respectively (Fig. 3b, c). In the multiparous sows of the two breeds, the numbers of TNB and NBA in sows with LAN were 1.43–1.51 and 1.63–2.70 higher than in sows with LAD, respectively (Fig. 3e, f). These results showed that LAD of sows would reduce the reproductive performance.

**Table 2:** Genetic correlations between LAD at different parities of sows

Population	Parity	Number	h^2^ ± SE	GC ± SE
Landrace	primiparous	487	0.12 ± 0.07	–
	multiparous	126	–	–
Yorkshire	primiparous	2776	0.11 ± 0.02	0.88 ± 0.08
	multiparous	857	0.19 ± 0.05	

h^2^ ± SE: Genomic heritability ± Standard error

GC ± SE: Genetic correlation ± Standard error

### Single-breed GWAS results

In this study, PCA was conducted to evaluate the population stratification of the two pig populations. The results of PCA showed that the Landrace and Yorkshire pigs were clustered in two different populations, indicating that the sows have different genetic backgrounds [Additional file 4, Fig. S2]. Thus, we conducted single-breed GWAS for LAD of Landrace and Yorkshire sows, respectively. Additionally, Q–Q plots revealed that there was no overall systematic bias in the LAD data for the two pig populations [Additional file 5, Fig. S3].

**Fig. 4:**
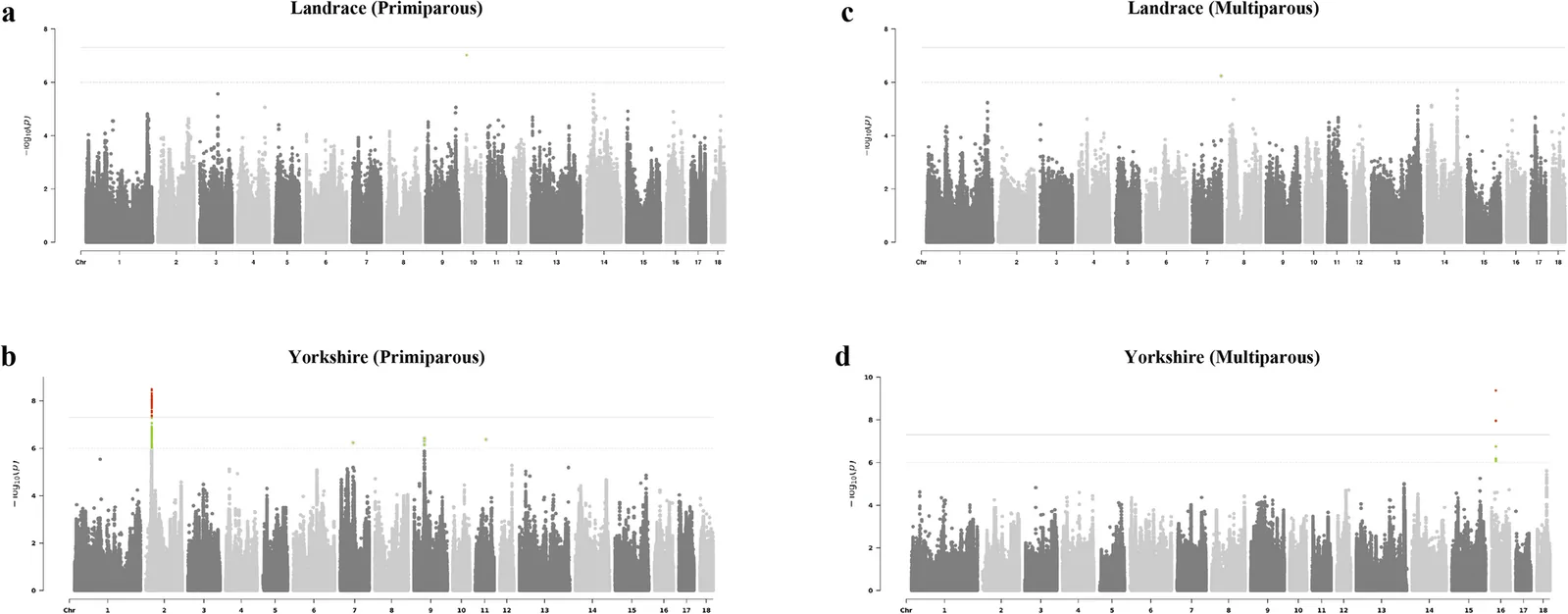
Manhattan plots of single-breed GWAS for LAD in sows. In the Manhattan plots, the solid and dashed lines represent the genome-wide and chromosome-wide (suggestive) thresholds, respectively. Manhattan plot for **a** Landrace (Primiparous); **b** Yorkshire (Primiparous); **c** Landrace (Multiparous); **d** Yorkshire (Multiparous)

For primiparous sows, one suggestive SNP (*P* < 1.00E-06) was detected to be associated with LAD in Landrace sows (Fig. 4a; Table 3). For Yorkshire sows, 249 SNPs were associated with LAD (Fig. 4b; Table 3), among which 90 SNPs reached genome-wide significance (*P* < 5.00E-08). Notably, 243 of these associated SNPs were located at approximately 23 Mb on SSC2 in Yorkshire sows (Fig. 4b; Table 3). These results suggested that the QTL on SSC2 might be the major QTL affecting LAD in sows. Additionally, a suggestive QTL was identified on SSC9 in Yorkshire primiparous sows, where four SNPs surpassed the suggestive significance threshold (Fig. 4b; Table 3). For multiparous sows, one suggestive SNP was detected to be associated with LAD in Landrace sows (Fig. 4c; Table 3). For Yorkshire sows, 11 SNPs were associated with LAD, among which three SNPs reached genome-wide significance (Fig. 4d; Table 3).

**Table 3:** Significant SNPs for LAD in different parities of sows

Pop^1^	Parity	SSC^2^	N^3^	M^4^	Position (Mb)^5^	Top SNP		
						SNP	*P*-value	PVE^6^ (%)
Landrace	Primiparous	10	1	0	7.64	10_7641070	9.52E-08	6.46
	Multiparous	7	1	0	119.18	7_119175130	5.74E-07	–
Yorkshire	Primiparous	2	243	90	22.63–23.76	2_23327820	3.21E-09	4.50
		7	1	0	54.42	7_54423840	5.86E-07	2.87
		9	4	0	43.66–44.12	9_43672208	3.87E-07	2.67
		11	1	0	44.81	11_44812843	4.24E-07	2.84
	Multiparous	16	11	3	18.92–19.36	16_18951943	4.23E-10	5.96

^1^Pop: Population

^2^SSC: *Sus scrofa* chromosome

^3^N: Number of significant SNPs (*P* < 1E-06)

^4^M: Number of significant SNPs (*P* < 5E-08)

^5^Position: Range of significants SNP in Ensembl

^6^PVE: Explained phenotypic variance

### Significant variant and enrichment analysis

All significant variants were annotated using the VEP tool. The results showed that a total of 12 functional genes were within or near the identified significant variants [Additional file 6, Table S3], Table 4). To evaluate whether these variants reside within functional regulatory regions, ChIP‑seq (H3K27ac, H3K4me3) and ATAC‑seq peak data from different pig breeds and tissues were downloaded from the publicly available GEO database (https://www.ncbi.nlm.nih.gov/geo/), and the overlap between our significant SNPs and these peaks was examined [Additional file 7, Table S4]. Kyoto Encyclopedia of Genes and Genomes (KEGG), Gene Ontology (GO) and National Human Genome Research Institute Genome-Wide Association Study Catalog (NHGRI GWAS Catalog) analyses were conducted to identify pathways and biological processes associated with LAD in sows. These analyses revealed that the candidate genes were enriched in pathways including regulation of lipid metabolism, extracellular matrix organization, cell adhesion and response to estrogen [Additional file 8, Table S5].

**Table 4:** Candidate genes for LAD in the single-breed GWASs

SSC^1^	Candidate genes	Position^2^	*N* ^3^	Consequence
2	*LOC100522921*	23.10–23.19	6	Downstream gene variant
	*LOC100523114*	23.68–23.76	15	Intron variant Downstream gene variant
	*LOC110259276*	23.32–23.43	182	Intron variant Upstream gene variant
7	*ACAN*	54.42	1	Upstream gene variant
	*HAPLN3*	54.42	1	Downstream gene variant
9	*LOC102159414*	43.66–43.67	3	Intron variant Downstream gene variant Non coding transcript variant
	*BUD13*	44.12	1	Intron variant Downstream gene variant
	*ZPR1*	44.12	1	Downstream gene variant
	*APOA1*	44.12	1	Downstream gene variant
	*APOC3*	44.12	1	Upstream gene variant
	*APOA4*	44.12	1	Downstream gene variant
	*APOA5*	44.12	1	Downstream gene variant
10	*GPATCH2*	7.64	1	Intron variant
11	*LOC102164197*	44.81	1	Intron variant
16	*NPR3*	18.92–18.97	5	Intron variant Downstream gene variant
	*LOC102162488*	18.92–19.36	11	Intron variant Upstream gene variant Non coding transcript variant
	*TARS1*	19.36	1	Upstream gene variant
	*TARS*	19.36	1	Upstream gene variant

^1^SSC: *Sus scrofa* chromosome

^2^Position: Range of significant SNPs in Ensembl

^3^N: Number of significant SNPs

### Regulatory region variants within major QTL on SSC2 identified by GWAS and fine-mapping

**Fig. 5:**
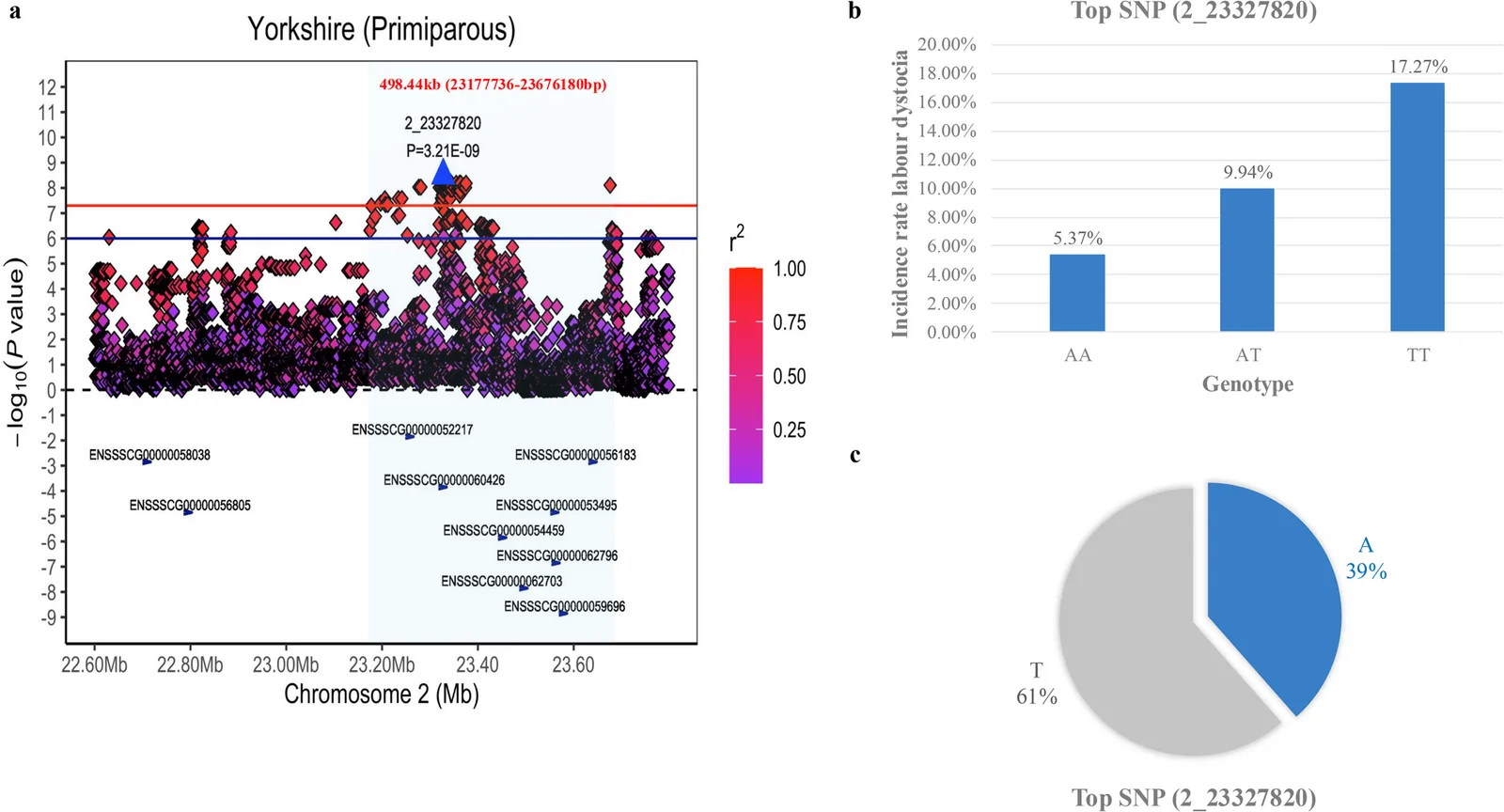
Regional association plot of the major QTL on SSC2. **a** The regional association plot shows the distribution of significant loci associated with LAD on SSC2. In the plot, the red and blue represent the genome-wide and chromosome-wide (suggestive) thresholds, respectively. A big blue triangle indicates the top SNP (2_23327820); **b** bar chart shows the incidence of LAD among primiparous Yorkshire sows with different genotypes of the top SNP (2_23327820); **c** Pie chart shows the frequencies of different alleles of the top SNP (2_23327820) in primiparous Yorkshire sows

In this study, a major QTL affecting LAD was identified on SSC2 of the primiparous Yorkshire sows. To determine the confidence interval of this QTL, the region was initially defined by all SNPs in strong LD (r² > 0.8) with the top SNP (2_23327820, *P* = 3.21E-09) and further refined using the 2-LOD drop-off method (i.e., all SNPs with a lod-score higher than the peak lod score minus 2 were retained). This fine-mapping procedure ultimately defined a 498.44 kb QTL interval (23.18–23.68 Mb) (Fig. 5a). The phenotypic variance explained by the top SNP (2_23327820) for the QTL region was 4.50% (Table 3). To investigate the effect of top SNP genotypes on LAD in primiparous Yorkshire sows, the incidence of LAD among sows with different genotypes of the top SNP was statistically analyzed. The results showed that Yorkshire sows with the AA genotype exhibited a lower incidence of LAD (5.37%) compared to those with AT (9.94%) and TT (12.27%) genotypes, and the frequency of the A allele was 39% (Fig. 5b–c). These findings suggested considerable potential for genetic improvement in the LAD trait of Yorkshire sows. We next performed Bayesian fine‑mapping using FINEMAP to investigate the number of independent causal signals at this locus. The model strongly rejected a single‑causal‑variant hypothesis (Pr (k = 1) = 0) and instead supported two independent causal signals (Pr (k = 2) = 0.645) [Additional file 9, Table S6]. Two 95% credible sets were identified: credible set 1 (cred1), led by 2_23327820 (PIP = 0.037), and credible set 2 (cred2), led by 2_23354970 (PIP = 0.222). The original 2‑LOD interval (498.44 kb, 23.18–23.68 Mb) served as the macroscopic boundary, within which these two credible sets were nested but spanned similarly broad ranges due to extremely high local linkage disequilibrium (LD). To further assess the possibility of additional independent signals, a conditional analysis was performed by including the top SNP (2_23327820) as a covariate in the mixed linear model. After conditioning on this SNP, all association signals within the QTL interval dropped below the significance threshold, suggesting that the detected effects are largely captured by the top SNP and its tightly linked variants [Additional file 10, Fig. S4]. This observation is consistent with the FINEMAP result that the region harbors at least two independent causal signals that are difficult to separate statistically due to strong LD.

**Fig. 6:**
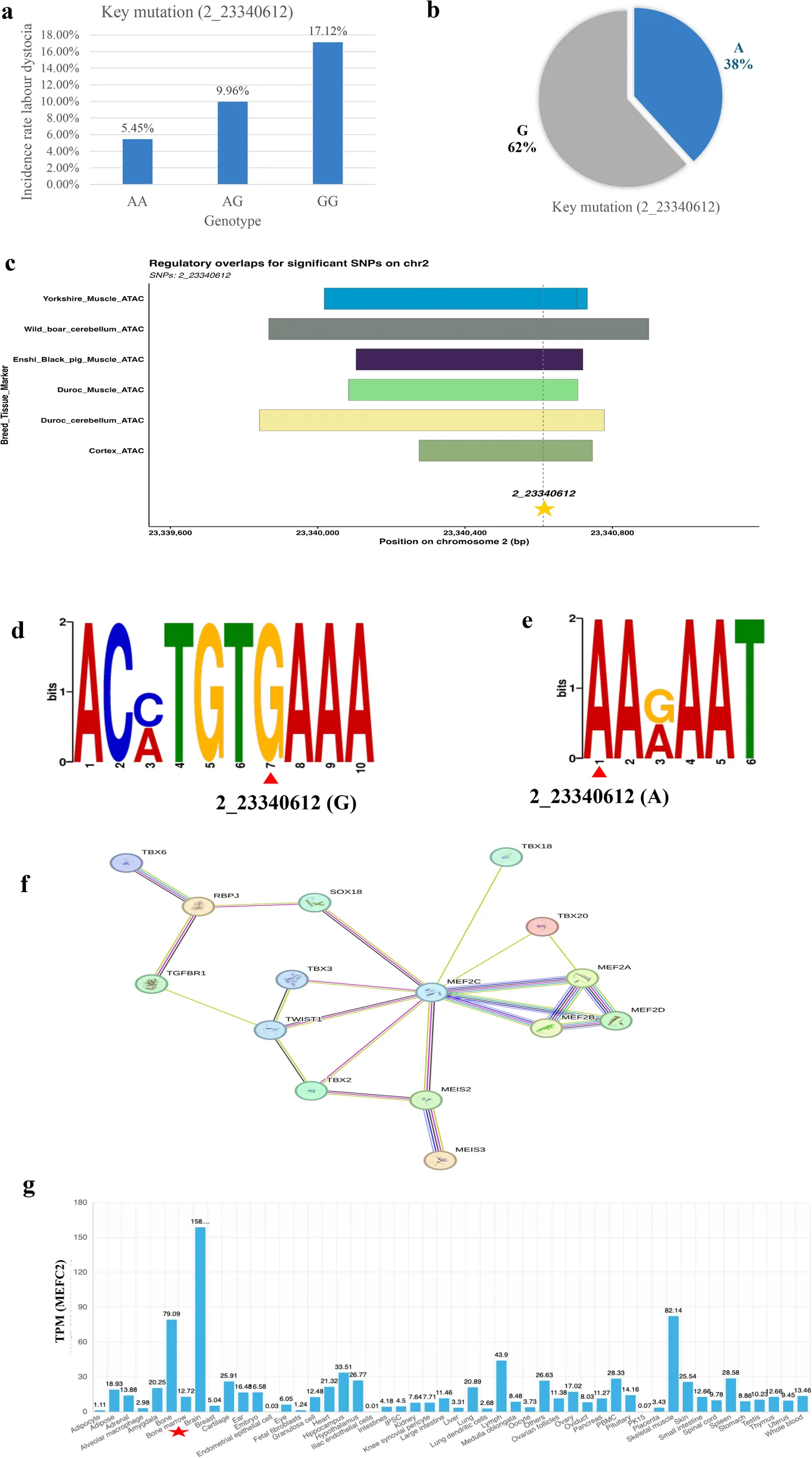
The motif analysis of a potentially regulatory SNP (2_23340612). **a** Bar chart shows the incidence of LAD among primiparity Yorkshire sows with different genotypes of the potentially regulatory SNP (2_23340612); **b** pie chart shows the frequencies of different alleles of potentially regulatory SNP (2_23340612) in primiparity Yorkshire sows; **c** schematic diagram of the overlap between the potentially regulatory SNP (2_23340612) and regulatory regions; **d** the A alleles of the potentially regulatory SNP (2_23340612) was located within the ACMTGTGAAA (motif one); **e** the G allele of the potentially regulatory SNP (2_23340612) was located within the AARAAT (motif two). The motifs were generated using the MEME Suite; **f** predicted interaction network among transcription factors potentially regulating the LAD trait in sows. Transcription factors are represented as nodes, and edges represent predicted interactions; **g** expression levels of *MEF2C* in different pig tissues as retrieved from the PIGOME website (https://pigome.com)

To assess the regulatory potential of the credible set variants, we examined their overlap with public ChIP‑seq (H3K27ac, H3K4me3) and ATAC‑seq peaks from diverse pig tissues. Among the SNPs of credible set, four SNPs (2_23340612, 2_23324582, 2_23324812, and 2_23325247) were annotated as residing within regulatory regions [Additional file 7, Table S4]. Of these, 2_23340612 (PIP = 0.012) overlapped with ATAC‑seq peaks from muscle and brain tissues of Yorkshire, Enshi black pig, and Duroc breeds, whereas the remaining three SNPs were exclusively annotated in brain‑derived regulatory regions of unknown pig breeds [Additional file 7, Table S4, Fig. 6c]. Based on this annotation, 2_23340612 was prioritized as a potentially regulatory variant for further investigation. As shown in Table 5, the SNP (2_23340612; *P* = 9.63E‑09) explained 4.29% of the phenotypic variance, and this SNP was in high LD with the top SNP (r^2^ = 0.99). In addition, the incidence of LAD among sows with different genotypes of this potentially regulatory SNP was statistically analyzed. The results showed that Yorkshire sows with the AA genotype exhibited a lower incidence of LAD (5.45%) compared to those with AG (9.96%) and GG (17.12%) genotypes, and the frequency of the A allele was 38% (Fig. 6a, b). Given that the SNP (2_23340612) is a potentially regulatory variant, the MEME Suite was used to predict whether this mutation is located in the TFBS. The results showed that alleles A and G of the potentially regulatory SNP (2_23340612) were located in ACMTGTGAAA (motif one) and AARAAT (motif two) motifs, respectively [Additional file 11, Table S7, Fig. 6d, e]. The TOMTOM online tool predicted 27 transcription factors [Additional file 11, Table S7]. For motifs, the KEGG pathway and GO term for transcription factors include female genitalia development, muscle contraction, Oxytocin signaling pathway, parturition [Additional file 12, Table S8]. Surprisingly, a network interaction map centered on the MEF2C transcription factor was identified in the STRING analysis, and this transcription factor was mainly involved in the oxytocin signalling pathway, muscle organ development, and DNA-binding transcription factor activity [Additional file 12, Table S8, Fig. 6f]. According to the PIGOME database (https://pigome.com), MEF2C is highly expressed in brain tissues (Fig. 6g). Although these observations are consistent with a potential regulatory role, the low posterior inclusion probability (PIP) of 2_23340612 in the FINEMAP analysis indicates that its causality remains uncertain. Therefore, the functional role of 2_23340612 requires further experimental investigation.

**Table 5:** Summary statistics of potentially regulatory SNPs on SSC2 and SSC9

SNP ID	*P*-value	PVE (%)^1^	*R*^2^ with top SNP
2_23340612	9.63E-09	4.29	0.99
9_44119733	3.95E-07	2.76	0.49

^1^PVE: Explained phenotypic variance

### Regulatory region variants within major QTL on SSC9 identified by GWAS and fine-mapping

**Fig. 7:**
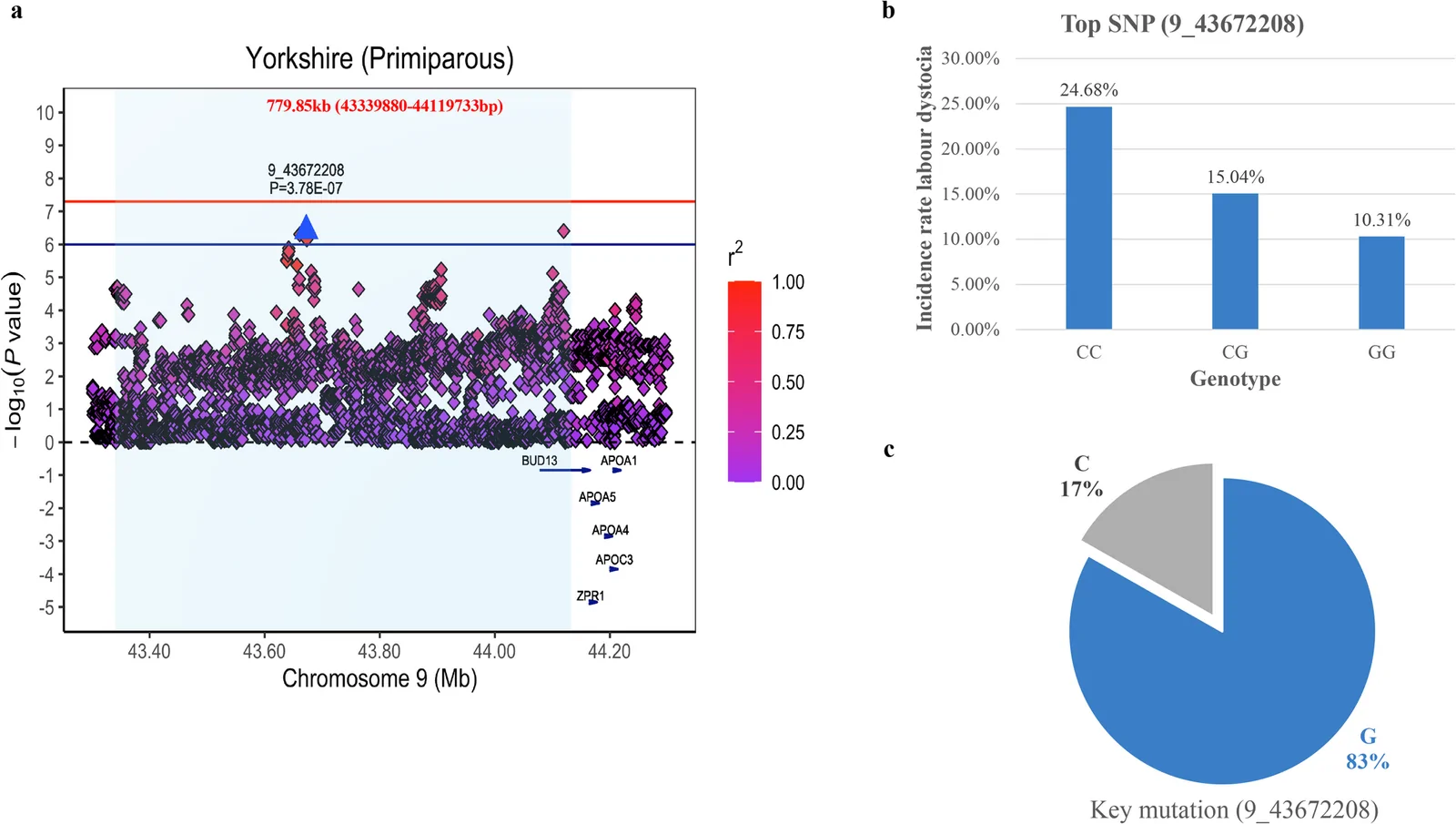
Regional association plot of the major QTL on SSC9. **a** The regional association plot shows the distribution of significant loci associated with LAD on SSC9. In the plot, the red and blue represent the genome-wide and chromosome-wide (suggestive) thresholds, respectively. A big blue triangle indicates the top SNP (9_43672208); **b** bar chart shows the incidence of LAD among primiparous Yorkshire sows with different genotypes of the top SNP (9_43672208); **c** pie chart shows the frequencies of different alleles of the top SNP (9_43672208) in primiparous Yorkshire sows

**Fig. 8:**
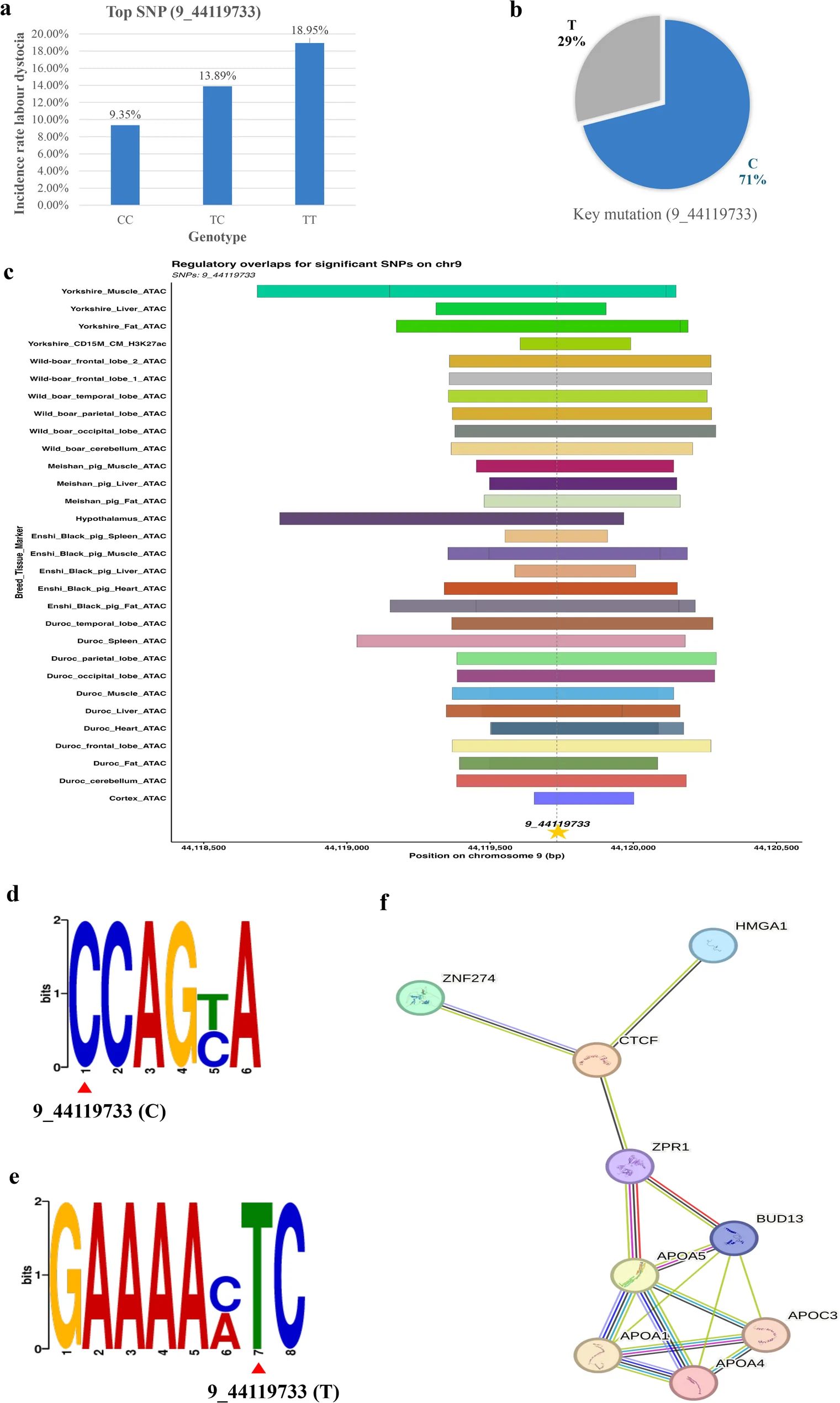
The motif analysis of a potentially regulatory SNP (9_4419733). **a** Bar chart shows the incidence of LAD among primiparity Yorkshire sows with different genotypes of the potentially regulatory SNP (9_4419733); **b** pie chart showed the frequencies of different alleles of potentially regulatory SNP (9_4419733) in primiparity Yorkshire sows; **c** schematic diagram of the overlap between potentially regulatory SNP (9_4419733) and regulatory regions; **d** the alleles C of potentially regulatory SNP (9_4419733) was located in CCAGYA (motif three); **e** The T of potentially regulatory SNP (9_4419733) was located in GAAAAMTC (motif four). The motifs were generated using MEME Suite; **f** Interaction network analysis of the candidate genes and transcription factors associated with LAD trait in sows. Candidate genes or transcription factors are represented as nodes, and the connecting lines represent interactions

On SSC9, a QTL for LAD was detected in Yorkshire primiparous sows. Given that only four SNPs on SSC9 surpassed the suggestive significance threshold, the QTL interval was defined by the 2‑LOD drop‑off method, spanning 779.85 kb (43.34–44.12 Mb) (Fig. 7a). As shown in Table 3, the top SNP (9_43672208) explained 2.67% of the phenotypic variance. Yorkshire sows carrying the CC genotype exhibited a higher incidence of LAD (24.68%) compared with those carrying the CG (15.04%) or GG (10.31%) genotype, and the frequency of the G allele was 83% (Fig. 7b, c). To further investigate the SSC9 QTL, all SNPs within this interval were subjected to Bayesian fine‑mapping using FINEMAP. The model assigned a posterior probability of 0.56 to the presence of two causal variants (Pr(k = 2)), whereas a single‑causal‑variant model received limited support (Pr(k = 1) = 0.034), and a three‑causal‑variant model also had a non‑negligible probability (Pr(k = 3) = 0.406). Two 95% credible sets were identified, with cred1 led by 9_44119733 (PIP = 0.089, the highest PIP in this interval), and cred2 led by 9_43680847 (PIP = 0.074) [Additional file 9, Table S6]. A conditional analysis was then performed by including the top SNP (9_43672208) as a covariate to test for additional independent signals. After conditioning on this SNP, all association signals within the QTL interval dropped below the significance threshold [Additional file 10, Fig. S4], indicating that the detected effects are largely captured by the top SNP and its tightly linked variants. Notably, this SNP overlaps with ATAC‑seq peaks in adipose, muscle, brain, heart, liver, and other tissues across multiple breeds, including Yorkshire, Duroc, Meishan, and Enshi black pig [Additional file 7, Table S4, Fig. 8c], suggesting that it resides within a conserved, active regulatory region. For SNP 9_44119733, the incidence of LAD also differed by genotype: sows with the TT genotype showed a higher incidence (18.95%) than those with the TC (13.89%) or CC (9.35%) genotypes, and the frequency of the C allele was 71% (Fig. 8a, b). As shown in Tables 5 and 9_44119733 explained 2.76% of the phenotypic variance, and the LD between this SNP and the top SNP was moderate (r² = 0.49).

Given that the SNP (9_44119733) is a regulatory region variant located in an intron of *BUD13* and within the upstream or downstream regions of *ZPR1*, *APOA1*, *APOC3*, *APOA4*, and *APOA5* [Additional file 6, Table S3], the MEME Suite was used to predict whether this mutation is located in TFBS. The results showed that alleles C and T of the potentially regulatory SNP (9_44119733) were located in CCAGYA (motif three) and GAAAAMTC (motif four) motifs, respectively ([Additional file 11, Table S7], Fig. 8d, e). The TOMTOM online tool predicted eight transcription factors [Additional file 11, Table S7]. For motifs, the KEGG pathway and GO term for transcription factors include DNA binding, chromatin binding, and RNA polymerase II, regulation of transcription [Additional file 12, Table S8]. Interestingly, an interaction network involving the transcription factors ZNF274, HMGA1, CTCF, and the candidate genes *ZPR1*, *APOA1*, *APOC3*, *APOA4*, *APOA5*, and *BUD13* was identified in the STRING analysis, and these molecules were mainly associated with transcriptional regulation, chromatin organization, and lipid‑related processes, etc. [Additional file 8, Table S5, Fig. 8f]. These observations suggest a potential regulatory network in which ZNF274, HMGA1, and CTCF interact with *ZPR1*, *APOA1*, *APOC3*, *APOA4*, *APOA5*, and *BUD13* to influence lipogenesis and lipid metabolism‑related pathways. However, whether such interactions occur in vivo and contribute to LAD remains to be experimentally determined.

## Discussion

### Effects of parity, breed, and piglet size on LAD

The incidence of LAD of sows was higher in the first parities (Landrace: 23.41%; Yorkshire: 12.03%), compared with that in the second to fourth parities (Landrace: 8.97–20.47%; Yorkshire: 8.97–10.91%) (Table 1). These results were similar to those of previous studies that first-parity dams had a higher probability of LAD than later-parity ones [42–44]. Research has shown that dam live weight was positively related to pelvic dimensions, with increasing pelvic conjugate diameter associated with a lower incidence of dystocia [45, 46]. Therefore, the authors speculated that the higher incidence of LAD in first-parity sows than in later ones resulted from some gilts being mated too early, as well as the sows’ live weights and pelvic development not meeting the requirements for mating. In this study, the incidence of LAD of the third and fourth parities sows significantly increased in comparison with the second parity. This phenomenon could be caused by the aging of the uterine muscle tissue and a reduction in the ability to contract after multiple deliveries, prolonging the parturient process and increasing the incidence of LAD in sows [9].

In this study, the incidence of LAD in Landrace and Yorkshire sows was 19.09% and 10.70%, respectively (Fig. 2a). It is worth noting that ABW of piglets born from Landrace and Yorkshire sows was1.58 kg and 1.33 kg, respectively (Fig. 2c). Many studies showed that larger piglets are more difficult to be expelled than smaller piglets because larger piglets have higher friction with the birth canal during delivery than smaller ones [10, 47]. These results suggest that the larger piglets may increase the incidence of LAD in sows. LAD can lead to uterine or vaginal prolapse, retained placenta or foetal tissue, and post-parturient ketosis, all of which harm the health of the sow [48, 49]. In this study, when the two population sows had LAD, the probability of LAD reappearance was 35.38% and 19.69% for Landrace and Yorkshire, respectively (Fig. 2b). The author speculated that LAD caused irreversible damage to sow health, which increases the recurrence of LAD in sows. In addition, as piglet size affects sow delivery, larger piglets may increase the recurrence rate of LAD in sows. In this study, LAD decreased the TNB and NBA significantly. These results indicated that LAD of sows was affected by breed, parity, and piglet size, and LAD has an inverse effect on sow health and piglet survival rate. Importantly, although the LAD trait is influenced by many factors, its heritability estimates were low to medium (from 0.11 to 0.19), indicating that genetic factors also contribute to this trait.

### Single-breed GWAS results

Sample size and case-control ratio were the main factors influencing the statistical power of GWAS. Generally, larger sample sizes and an optimal case-control ratio enhance the statistical power of GWAS. However, many GWAS studies collected samples from populations with different genetic backgrounds, as population stratification is the main cause of false positive results in GWAS [50]. To eliminate population stratification, many studies have conducted single-breed GWASs for different populations [51, 52]. Single-breed GWAS can eliminate the influence of population stratification to a large extent, and the many specific SNPs can be retained in the quality control of SNPs.

Given that PCA revealed distinct genetic backgrounds between Landrace and Yorkshire pig populations, we performed single-breed GWAS for the LAD trait separately in primiparous and multiparous sows. The sample sizes ranged from 126 to 487 for Landrace and from 857 to 2776 for Yorkshire [Additional file 2, Table S1]. Correspondingly, the number of significant SNPs detected in Landrace sows was substantially lower than in Yorkshire sows. Specifically, we identified one and 250 associated SNPs in primiparous Landrace and Yorkshire sows, and one and 11 SNPs in their multiparous counterparts, respectively (Table 3; Fig. 4). This disparity indicates that statistical power was significantly affected by sample size. Furthermore, no significant SNPs overlapped between the primiparous and multiparous sow analyses, suggesting that the case-control ratio may also be an influential factor. This observation aligns with the simulation study by Turgut et al. [53], which demonstrated that increasing the sample size can enhance GWAS power when cases are limited, and that a case/control ratio of 1:1 is the optimal ratio for GWAS.

Additionally, this study combined the GWAS results from the Landrace and Yorkshire populations in a meta-analysis to validate the single-breed GWAS findings. As shown in the [Additional file 13, Fig. S5], for primiparous sows, the meta-analysis identified 167 significant SNPs, fewer than the 250 significant SNPs found in the single-breed GWASs, with only 2 novel SNPs. For multiparous sows, the meta-analysis detected no significant signals, whereas the single-breed GWASs identified 12 significant SNPs. The meta-analysis results indicate significant genetic heterogeneity across breeds. The effects likely differ between Yorkshire and Landrace populations, diluting combined signals. Notably, a major QTL affecting the LAD trait was detected on SSC2 in primiparous Yorkshire sows. This QTL was also identified in the meta-analysis, corroborating that it is not a false positive.

### Candidate genes and transcription factor

A total of 12 functional genes were determined within or near the significant SNPs in this study. Further gene enrichment analysis was performed on these 12 genes, among which eight genes were selected as candidate genes for LAD occurrence, including Apolipoprotein A1 (*APOA1*), Apolipoprotein C3 (*APOC3*), Apolipoprotein A4 (*APOA4*), Apolipoprotein A5 (*APOA5*), BUD13 Spliceosome Associated Protein (*BUD13*), ZPR1 Zinc Finger (*ZPR1*), Aggrecan (*ACAN*), Hyaluronan And Proteoglycan Link Protein 3 (*HAPLN3*).

In this study, one significant SNP (7_54423840) was identified as a variant located upstream of *ACAN* and downstream of *HAPLN3*. GO analysis showed that the *ACAN* and *HAPLN3* genes were involved in extracellular matrix and cell adhesion. The extracellular matrix and cell adhesion pathways are essential components of animal parturition. They coordinate processes such as myometrial contraction, cervical softening, and placental remodelling, ensuring successful gestation and smooth labor progression [54–56]. Thus, *ACAN* and *HAPLN3* likely play important roles in parturition. Additionally, one significant SNP (9_44119733) was annotated as an upstream/downstream variant of six genes (*APOA1*, *APOC3*, *APOA4*, *APOA5*, *BUD13*, and *ZPR1*). KEGG, GO, and NHGRI GWAS Catalog analysis showed that the six genes participate in lipid metabolism, such as fat digestion and absorption, lipoprotein metabolic process, positive regulation of lipid biosynthetic process, etc. Notably, population‑scale studies have identified *BUD13*, *ZPR1*, and *APOA5* as being significantly associated with lipid metabolite levels and metabolic health [57], further supporting the role of these genes in lipid metabolism. Many studies have shown that high backfat (> 21 mm) in sows leads to reduced movement during delivery and prolonged delivery time, while low backfat (< 16 mm) may lead to insufficient energy reserve of sows, increasing the incidence of LAD [3, 58, 59]. Therefore, these six genes (*APOA1*, *APOC3*, *APOA4*, *APOA5*, *BUD13*, and *ZPR1*) may influence sow parturition through lipid metabolism. Notably, GO analysis indicated *APOA1* gene involvement in the estrogen response, transforming growth factor beta (TGF-β) receptor, and integrin-mediated signalling pathways. Studies indicate that estrogen, the TGF-β receptor, and integrin-mediated signalling pathways play pivotal roles in the process of parturition by regulating uterine smooth muscle function, cervical ripening, and extracellular matrix remodelling, thereby influencing the progression of labor [60–62]. Thus, the *APOA1* gene could be considered a strong candidate gene for LAD. Notably, the potentially regulatory SNP (9_44119733) overlaps with ATAC‑seq peaks in adipose, muscle, brain, heart, liver, and other tissues across multiple breeds, including Yorkshire, Duroc, Meishan, and Enshi black pig [Additional file 7, Table S4, Fig. 8c], suggesting that it resides within a conserved, active regulatory region. Furthermore, transcription factor binding site prediction showed that the potentially regulatory SNP (9_44119733) resides within motifs recognized by transcription factors such as ZNF274, HMGA1, CTCF, ZNF157, GCM2, and ONECUT3. STRING analysis further predicted interactions among ZNF274, HMGA1, CTCF, and the neighboring genes *APOA1*, *APOC3*, *APOA4*, *APOA5*, *BUD13*, and *ZPR1*, suggesting a regulatory network associated with transcriptional regulation, chromatin organization, and lipid‑related processes. Therefore, we speculate that ZNF274, HMGA1, and CTCF may interact with *APOA1*, *APOC3*, *APOA4*, *APOA5*, *BUD13*, and *ZPR1* to jointly influence lipogenesis and lipid metabolism‑related pathways. However, this hypothesis requires further experimental validation.

The potentially regulatory SNP (2_23340612) for the LAD trait identified in this study was located in the ACMTGTGAAA (motif one) and AARAAT (motif two) motifs, which together were predicted to bind a total of 27 transcription factors. Interaction network analysis of these 27 transcription factors using STRING software revealed a network centered on the MEF2C transcription factor (Fig. 6f). KEGG and GO analysis showed that the MEF2C was mainly involved in the oxytocin signalling pathway, muscle organ development, and DNA-binding transcription factor activity, etc. [Additional file 12, Table S8]. Oxytocin, a nonapeptide hormone, is primarily synthesized in the hypothalamus and subsequently released into circulation via the posterior pituitary [63]. Notably, this potentially regulatory SNP (2_23340612) was located within open chromatin regions in muscle and brain tissues from the Yorkshire, Enshi black pig, and Duroc breeds (Fig. 6c). It may function as an MEFC2 transcription factor binding site involved in regulating the synthesis and secretion of oxytocin in the hypothalamus and pituitary gland. Moreover, GO analysis revealed that transcription factors interacting with MEF2C were involved in embryonic development, female genitalia development, muscle contraction, and related biological processes. It is well known that uterine development, uterine contractions, and fetal size collectively influence sow parturition. Generally, uterine malformation, insufficient uterine contractions, and excessive fetal size can increase the risk of LAD in female animals [10, 47, 64]. Although these observations are consistent with a potential regulatory role, the low posterior inclusion probability (PIP) of 2_23340612 in the FINEMAP analysis indicates that its causality remains uncertain. Therefore, the functional role of 2_23340612 and its proposed interaction with MEF2C require further experimental investigation.

## Conclusions

In this study, we found that LAD could reduce piglet survival rate and affect the reproductive performance of the sow. In addition, parity, breed, and piglet size could influence the incidence of LAD in sows. The single-breed GWASs identified 262 SNPs that were associated with LAD, among which potentially regulatory SNPs (2_23340612 and 9_44119733) and eight genes (*APOA1*, *APOC3*, *APOA4*, *APOA5*, *BUD13*, *ZPR1*, *ACAN*, *HAPLN3*) were prioritized for their potential relevance to LAD occurrence. Notably, SNP 9_44119733 overlaps with ATAC‑seq peaks in adipose, muscle, brain, heart, liver, and other tissues across multiple breeds, suggesting that it resides within a conserved active regulatory region; transcription factor binding site prediction further indicated that this SNP may interact with ZNF274, HMGA1, and CTCF, which together with the neighboring genes form a regulatory network associated with lipid metabolism‑related pathways. However, this hypothesis requires further experimental validation. Bayesian fine‑mapping further indicated that the SSC2 and SSC9 QTLs likely harbour at least two independent causal signals, and no single SNP could be designated as the sole causal variant. Therefore, these variants and genes should be regarded as candidate loci requiring further functional validation. Given that humans are very similar to swine in terms of anatomy, physiology, and the delivery process, these findings may provide a reference for LAD research in both species.

## Supplementary Information

Below is the link to the electronic supplementary material.

Supplementary Material 1: Imputation accuracy of filtered variants across autosomes. Boxplots show the distribution of imputation accuracy, measured as dosage R2 (DR²), for variants passing the quality filter on each chromosome. The central line within each box represents the median DR² value, while the box and whiskers span the interquartile range and the full range of observed values, respectively. Red numerical values above each box denote the mean imputation accuracy (mean DR²) for variants on the corresponding chromosome.

Supplementary Material 2: Statistics on the number of effective SNPs in different populations. Number of Cases: Number of sows with LAD; Number of Controls: Number of sows with LAN.

Supplementary Material 3: Genetic correlations between LAD and reproductive traits in sows. Trait: Labour dystocia (LAD); Total number born (TNB); Number born alive (NBA).

Supplementary Material 4: PCA plot of population structure showing the top two principal components. pc1: principal component 1; pc2: principal component 2.

Supplementary Material 5: Q–Q plots showing the observed versus expected log10(P-values) for LAD by grouping method one.

Supplementary Material 6: Results of significant SNPs annotation.

Supplementary Material 7: Overlap of significant SNPs with ChIP‑seq and ATAC‑seq peaks across pig breeds and tissues. ChIP-seq peaks represent histone modifications (H3K27ac and H3K4me3); ATAC-seq peaks indicate chromatin accessibility. All data were retrieved from the public GEO database (https://www.ncbi.nlm.nih.gov/geo/). Complete sample names and corresponding GEO accession numbers are provided in this Additional file.

Supplementary Material 8: Results of KEGG, GO, and NHGRI GWAS Catalog analysis for candidate genes.

Supplementary Material 9: FINEMAP 95% credible sets for the SSC2 and SSC9 QTLs.

Supplementary Material 10: Regional Manhattan plots of GWAS results before and after conditional analysis for primiparous Yorkshire pigs. (a) Results for the SSC2 region (22.6–23.8 Mb). The top panel shows the original GWAS associations, with the top SNP at 2:23327820 (P = 3.21E-09) marked. The bottom panel shows associations after conditional analysis on the top SNP; (b) Results for the SSC9 region (43.3–44.2 Mb). The top panel shows the original associations, with the top SNP at 9:43672208 (P = 3.78E-07) marked. The bottom panel shows associations after conditional analysis on the top SNP. Points are coloured according to their linkage disequilibrium (R²) with the top SNP, ranging from blue (R² = 0) to red (R² = 1.00). The red and blue dashed lines indicate the genome-wide significance (P = 5.00E-08) and suggestive significance (P = 1.00E-06) thresholds, respectively.

Supplementary Material 11: Results of the predicted motif and transcription factors.

Supplementary Material 12: Results of KEGG and GO analysis for the predicted transcription factors.

Supplementary Material 13: Meta-analysis of GWAS and Venn plot of associated SNPs.

## Data Availability

The datasets generated and/or analysed during the current study are available in the Figshare repository: https://figshare.com/s/51ece5c2f5cfa2637dc5.
