# Supplementary material for: Imputed whole genome sequence based association pinpoints genetic architecture of labour dystocia of pigs using multi-breed populations
Source: Genet Sel Evol. 2026 Aug 9;58:58. doi: 10.1186/s12711-026-01076-3 (PMC13501852; doi:10.1186/s12711-026-01076-3)
Supplement: Supplementary file 1 [file 12711_2026_1076_MOESM2_ESM.docx]

**Additional file 2:** Table S1. Statistics on the number of effective SNPs in different populations

| **Population** | **Parity** | **Number of Cases^1^** | **Number of Controls^2^** | **Effective SNPs** |
| --- | --- | --- | --- | --- |
| Landrace | Primiparity | 114 | 373 | 7,134,343 |
|  | Multiparity | 46 | 80 | 6,976,227 |
| Yorshire | Primiparity | 334 | 2442 | 7,151,715 |
|  | Multiparity | 110 | 747 | 7,138,971 |

^1^Number of Case: Number of sows with LAD;

^2^Number of Control: Number of sows with LAN;
