# Supplementary material for: Imputed whole genome sequence based association pinpoints genetic architecture of labour dystocia of pigs using multi-breed populations
Source: Genet Sel Evol. 2026 Aug 9;58:58. doi: 10.1186/s12711-026-01076-3 (PMC13501852; doi:10.1186/s12711-026-01076-3)
Supplement: Supplementary file 2 [file 12711_2026_1076_MOESM3_ESM.docx]

**Additional file 3:** Table S2. Genetic correlation between LAD and reproductive traits in sows

| **Trait^1^** | **Genetic correlation** |
| --- | --- |
| LAD-TNB | -0.77 |
| LAD-NBA | -0.89 |

^1^Trait: Labour dystocia (LAD); Total number born (TNB); Number born alive (NBA)
