# Supplementary material for: Imputed whole genome sequence based association pinpoints genetic architecture of labour dystocia of pigs using multi-breed populations
Source: Genet Sel Evol. 2026 Aug 9;58:58. doi: 10.1186/s12711-026-01076-3 (PMC13501852; doi:10.1186/s12711-026-01076-3)
Supplement: Supplementary file 3 [file 12711_2026_1076_MOESM8_ESM.docx]

**Additional file 8.** Table S5. Results of gene enrichment analysis

| **Term** | **Database^1^** | **ID** | ***P*-Value** | **Gene** |
| --- | --- | --- | --- | --- |
| Fat digestion and absorption | KEGG PATHWAY | hsa04975 | 2.50E-04 | *APOA4; APOA1* |
| PPAR signaling pathway | KEGG PATHWAY | hsa03320 | 1.58E-05 | *APOA5; APOC3; APOA1* |
| lipoprotein metabolic process | Gene Ontology | GO:0042157 | 1.04E-09 | *APOA4; APOA5; APOC3; APOA1* |
| positive regulation of lipid biosynthetic process | Gene Ontology | GO:0046889 | 1.14E-07 | *APOA4*; *APOA5*; *APOA1* |
| lipase inhibitor activity | Gene Ontology | GO:0055102 | 1.02E-05 | *APOC3; APOA1* |
| collagen-containing extracellular matrix | Gene Ontology | GO:0062023 | 1.24E-05 | *APOA4; ACAN; APOC3; APOA1* |
| triglyceride metabolic process | Gene Ontology | GO:0006641 | 1.68E-04 | *APOA5; APOC3* |
| lipid binding | Gene Ontology | GO:0008289 | 2.66E-03 | *APOA4; APOA5* |
| extracellular matrix | Gene Ontology | GO:0031012 | 5.18E-03 | *ACAN*; *HAPLN3* |
| extracellular matrix structural constituent conferring compression resistance | Gene Ontology | GO:0030021 | 9.59E-03 | *ACAN* |
| cell adhesion | Gene Ontology | GO:0007155 | 1.31E-02 | *ACAN*; *HAPLN3* |
| response to estrogen | Gene Ontology | GO:0043627 | 2.33E-02 | *APOA1* |
| transforming growth factor beta receptor signaling pathway | Gene Ontology | GO:0007179 | 3.35E-02 | *APOA1* |
| integrin-mediated signaling pathway | Gene Ontology | GO:0007229 | 3.37E-02 | *APOA1* |
| Hypertriglyceridemia | NHGRI GWAS Catalog | - | 4.14E-15 | *BUD13; ZPR1; APOC3; APOA4; APOA5; APOA1* |
| Lipid traits | NHGRI GWAS Catalog | - | 2.59E-11 | *APOA4; APOA5; BUD13; APOC3; APOA1* |
| Triglycerides | NHGRI GWAS Catalog | - | 2.59E-11 | *BUD13; ZPR1 APOC3 APOA4 APOA5 APOA1* |
